# 1*H*-Benzotriazole–4-hy­droxy­benzoic acid (1/1)

**DOI:** 10.1107/S1600536813026767

**Published:** 2013-10-02

**Authors:** A. Thirunavukkarasu, A. Silambarasan, G. Chakkaravarthi, R. Mohankumar, P. R. Umarani

**Affiliations:** a Department of Physics, Presidency College, Chennai 600 005, India; b Department of Physics, CPCL Polytechnic College, Chennai 600 068, India; c Department of Physics, Presidency College, Chennai 600 005, India; d Kunthavai Naacchiyaar Govt. Arts College (W), Thanjavur 613 007, India

## Abstract

The asymmetric unit of the title compound, C_6_H_5_N_3_·C_7_H_6_O_3_, comprises independent benzotriazole and 4-hydroxybenzoic acid molecules. The dihedral angle between the benzene ring and the benzotriazole ring system is 15.18 (7)°. The mean plane of the carb­oxyl group is twisted at an angle of 18.55 (1)° with respect to the benzene ring. The crystal structure is stabilized by weak inter­molecular N—H⋯N, O—H⋯N, O—H⋯O and C—H⋯O inter­actions, forming a three-dimensional network.

## Related literature

For biological activities of benzotriazole derivates, see: Dubey *et al.* (2011 ▶); Gaikwad, *et al.* (2012 ▶). For reported structures, see: Sieroń (2007 ▶); Sudhahar *et al.*(2013 ▶); Yang *et al.* (2010 ▶).
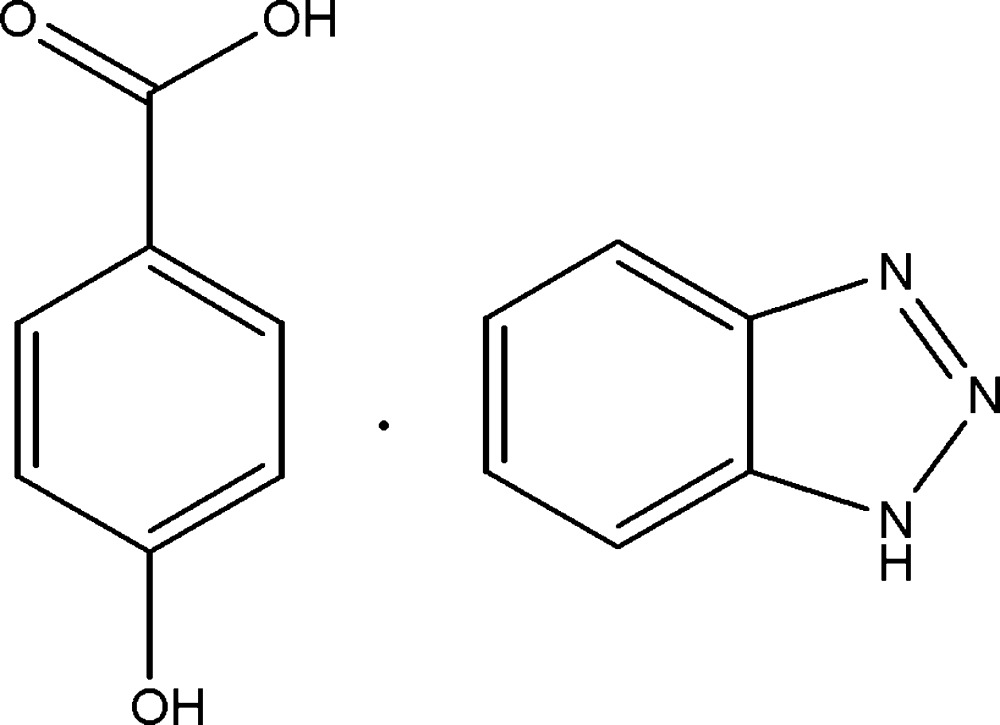


## Experimental

### 

#### Crystal data

C_6_H_5_N_3_·C_7_H_6_O_3_
*M* *_r_* = 257.25
Orthorhombic, 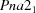

*a* = 17.3634 (13) Å
*b* = 11.4669 (9) Å
*c* = 6.0818 (4) Å
*V* = 1210.91 (15) Å^3^
*Z* = 4
Mo *K*α radiation
μ = 0.10 mm^−1^
*T* = 295 K
0.30 × 0.26 × 0.24 mm

#### Data collection

Bruker Kappa APEXII CCD diffractometer
Absorption correction: multi-scan (*SADABS*; Sheldrick, 1996 ▶) *T*_min_ = 0.970, *T*_max_ = 0.976
6611 measured reflections
2195 independent reflections
1925 reflections with *I* > 2σ(*I*)
*R*_int_ = 0.033

#### Refinement

*R*[*F* ^2^ > 2σ(*F* ^2^)] = 0.031
*wR*(*F* ^2^) = 0.074
*S* = 1.04
2195 reflections
174 parameters
1 restraint
H-atom parameters constrained
Δρ_max_ = 0.15 e Å^−3^
Δρ_min_ = −0.11 e Å^−3^

### 

Data collection: *APEX2* (Bruker, 2004 ▶); cell refinement: *SAINT* (Bruker, 2004 ▶); data reduction: *SAINT*; program(s) used to solve structure: *SHELXS97* (Sheldrick, 2008 ▶); program(s) used to refine structure: *SHELXL97* (Sheldrick, 2008 ▶); molecular graphics: *PLATON* (Spek, 2009 ▶); software used to prepare material for publication: *SHELXL97*.

## Supplementary Material

Crystal structure: contains datablock(s) global, I. DOI: 10.1107/S1600536813026767/vm2200sup1.cif

Structure factors: contains datablock(s) I. DOI: 10.1107/S1600536813026767/vm2200Isup2.hkl

Supplementary material file. DOI: 10.1107/S1600536813026767/vm2200Isup3.cml

Additional supplementary materials:  crystallographic information; 3D view; checkCIF report

## Figures and Tables

**Table 1:** Hydrogen-bond geometry (Å, °)

*D*—H⋯*A*	*D*—H	H⋯*A*	*D*⋯*A*	*D*—H⋯*A*
N1—H1⋯N2^i^	0.86	2.20	2.982 (2)	152
O2—H2*A*⋯N3^ii^	0.82	1.87	2.6817 (19)	169
O3—H3*A*⋯O1^iii^	0.82	1.88	2.6912 (17)	171
C13—H13⋯O3^iv^	0.93	2.49	3.373 (2)	159

Symmetry codes: (i) 
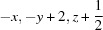
; (ii) 
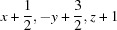
; (iii) 
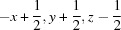
; (iv) 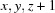.

**Fig. 1.**
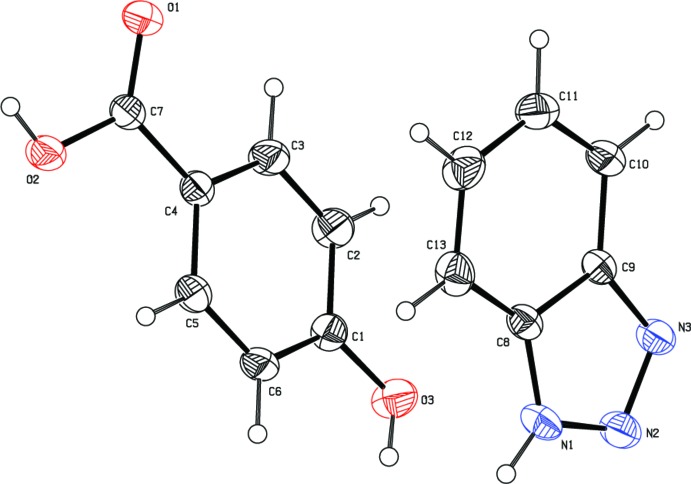
The molecular structure of (I), with atom labels and 30% probability displacement ellipsoids for non-H atoms.

**Fig. 2.**
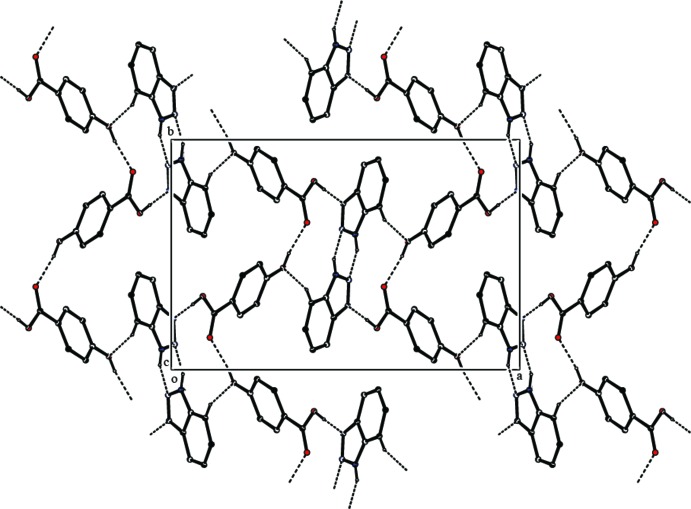
The packing of (I), viewed down c axis. Intermolecular hydrogen bonds are shown as dashed lines. H atoms not involved in hydrogen bonding have been omitted.

## supplementary crystallographic information

### 1. Comment

Benzotriazole derivates exhibit numerous essential bioactivitities, especially in antitubercular and antimicrobial (Dubey *et al.*, 2011; Gaikwad *et al.*, 2012) activities. We herewith report the crystal structure of the title compound (I) (Fig.1). The geometric parameters are comparable with reported structures (Sieroń , 2007; Sudhahar *et al.*, 2013; Yang *et al.*, 2010).

The benzene ring (C1-C6) is planar, with the maximum deviation of 0.010 (2) Å. The dihedral angle between the benzene ring and benzotriazole ring system is 15.18 (7)°. The mean plane of carboxyl group is twisted at an angle of 18.55 (1)° with the benzene ring. The crystal structure is stabilized by weak intermolecular N—H···N, O—H···N, O—H···O, C—H···O (Table 1 & Fig. 2) interactions to form a three dimensional network.

### 2. Experimental

Benzotriazole (C_6_H_5_N_3_, 1.1913 g) and p-hydroxy benzoic acid (C_7_H_6_O_3_, 1.3812 g) were mixed in equimolar ratio in methanol and the prepared solution was allowed for slow evaporation at room temperature. Good quality crystals suitable for X-ray intensity data collection were collected in a period of 10 days.

### 3. Refinement

H atoms were positioned geometrically and refined using riding model with C-H = 0.93 Å and U_iso_(H) = 1.2U_eq_(C) for CH, N-H = 0.86 Å and U_iso_(H) = 1.2U_eq_(C) for NH, O-H = 0.82 Å and U_iso_(H) = 1.5U_eq_(C) for OH.

### Crystal data

**Table:**

C_6_H_5_N_3_·C_7_H_6_O_3_	*F*(000) = 536
*M**_r_* = 257.25	*D*_x_ = 1.411 Mg m^−^^3^
Orthorhombic, *P**n**a*2_1_	Mo *K*α radiation, λ = 0.71073 Å
Hall symbol: P 2c -2n	Cell parameters from 2578 reflections
*a* = 17.3634 (13) Å	θ = 2.1–25.2°
*b* = 11.4669 (9) Å	µ = 0.10 mm^−^^1^
*c* = 6.0818 (4) Å	*T* = 295 K
*V* = 1210.91 (15) Å^3^	Block, colourless
*Z* = 4	0.30 × 0.26 × 0.24 mm

### Data collection

**Table:**

Bruker Kappa APEXII CCD diffractometer	2195 independent reflections
Radiation source: fine-focus sealed tube	1925 reflections with *I* > 2σ(*I*)
Graphite monochromator	*R*_int_ = 0.033
ω and φ scan	θ_max_ = 26.8°, θ_min_ = 2.1°
Absorption correction: multi-scan (*SADABS*; Sheldrick, 1996)	*h* = −20→22
*T*_min_ = 0.970, *T*_max_ = 0.976	*k* = −13→14
6611 measured reflections	*l* = −7→4

### Refinement

**Table:**

Refinement on *F*^2^	Secondary atom site location: difference Fourier map
Least-squares matrix: full	Hydrogen site location: inferred from neighbouring sites
*R*[*F*^2^ > 2σ(*F*^2^)] = 0.031	H-atom parameters constrained
*wR*(*F*^2^) = 0.074	*w* = 1/[σ^2^(*F*_o_^2^) + (0.0362*P*)^2^ + 0.0577*P*] where *P* = (*F*_o_^2^ + 2*F*_c_^2^)/3
*S* = 1.04	(Δ/σ)_max_ < 0.001
2195 reflections	Δρ_max_ = 0.15 e Å^−^^3^
174 parameters	Δρ_min_ = −0.11 e Å^−^^3^
1 restraint	Extinction correction: *SHELXL97* (Sheldrick, 2008), Fc^*^=kFc[1+0.001xFc^2^λ^3^/sin(2θ)]^-1/4^
Primary atom site location: structure-invariant direct methods	Extinction coefficient: 0.025 (2)

### Special details

**Table:**

Geometry. All esds (except the esd in the dihedral angle between two l.s. planes) are estimated using the full covariance matrix. The cell esds are taken into account individually in the estimation of esds in distances, angles and torsion angles; correlations between esds in cell parameters are only used when they are defined by crystal symmetry. An approximate (isotropic) treatment of cell esds is used for estimating esds involving l.s. planes.
Refinement. Refinement of F^2^ against ALL reflections. The weighted R-factor wR and goodness of fit S are based on F^2^, conventional R-factors R are based on F, with F set to zero for negative F^2^. The threshold expression of F^2^ > 2sigma(F^2^) is used only for calculating R-factors(gt) etc. and is not relevant to the choice of reflections for refinement. R-factors based on F^2^ are statistically about twice as large as those based on F, and R- factors based on ALL data will be even larger.

### Fractional atomic coordinates and isotropic or equivalent isotropic displacement parameters (Å^2^)

**Table:**

	*x*	*y*	*z*	*U*_iso_*/*U*_eq_	
C7	0.37669 (8)	0.74471 (13)	0.7594 (3)	0.0363 (4)	
C4	0.32444 (9)	0.79794 (14)	0.5984 (3)	0.0353 (4)	
C3	0.30244 (10)	0.73727 (15)	0.4112 (3)	0.0457 (4)	
H3	0.3204	0.6617	0.3895	0.055*	
C2	0.25482 (10)	0.78675 (16)	0.2581 (3)	0.0492 (5)	
H2	0.2416	0.7452	0.1323	0.059*	
C1	0.22618 (9)	0.89793 (15)	0.2887 (3)	0.0385 (4)	
C6	0.24605 (9)	0.95891 (15)	0.4762 (3)	0.0417 (4)	
H6	0.2261	1.0331	0.4997	0.050*	
C5	0.29535 (10)	0.91033 (14)	0.6286 (3)	0.0419 (4)	
H5	0.3093	0.9528	0.7527	0.050*	
N2	−0.00946 (8)	0.88952 (13)	0.2498 (3)	0.0477 (4)	
C9	0.03383 (9)	0.72630 (14)	0.3824 (3)	0.0345 (4)	
C8	0.05616 (10)	0.81341 (14)	0.5280 (3)	0.0366 (4)	
C13	0.09730 (10)	0.78968 (17)	0.7199 (3)	0.0485 (5)	
H13	0.1117	0.8480	0.8178	0.058*	
C12	0.11490 (10)	0.67520 (17)	0.7544 (4)	0.0539 (5)	
H12	0.1419	0.6551	0.8809	0.065*	
C11	0.09398 (12)	0.58653 (16)	0.6071 (3)	0.0521 (5)	
H11	0.1083	0.5101	0.6375	0.062*	
C10	0.05347 (10)	0.60939 (14)	0.4216 (3)	0.0459 (4)	
H10	0.0393	0.5504	0.3248	0.055*	
N3	−0.00650 (8)	0.77773 (12)	0.2146 (2)	0.0418 (3)	
N1	0.02792 (8)	0.91193 (12)	0.4377 (3)	0.0456 (4)	
H1	0.0332	0.9803	0.4935	0.055*	
O1	0.38942 (6)	0.63969 (10)	0.7712 (2)	0.0472 (3)	
O2	0.40973 (7)	0.81933 (10)	0.8925 (2)	0.0504 (3)	
H2A	0.4380	0.7843	0.9779	0.076*	
O3	0.17907 (8)	0.94246 (11)	0.13231 (19)	0.0531 (4)	
H3A	0.1627	1.0060	0.1729	0.080*	

### Atomic displacement parameters (Å^2^)

**Table:**

	*U*^11^	*U*^22^	*U*^33^	*U*^12^	*U*^13^	*U*^23^
C7	0.0366 (8)	0.0325 (9)	0.0398 (9)	−0.0021 (6)	0.0031 (7)	−0.0020 (9)
C4	0.0346 (8)	0.0338 (8)	0.0376 (9)	−0.0022 (6)	0.0022 (7)	−0.0032 (7)
C3	0.0509 (10)	0.0377 (9)	0.0485 (11)	0.0083 (7)	−0.0051 (9)	−0.0119 (10)
C2	0.0575 (10)	0.0467 (10)	0.0434 (11)	0.0084 (8)	−0.0093 (10)	−0.0186 (9)
C1	0.0391 (8)	0.0405 (9)	0.0359 (9)	0.0005 (7)	−0.0001 (7)	−0.0029 (8)
C6	0.0480 (9)	0.0303 (8)	0.0468 (10)	0.0029 (7)	−0.0041 (8)	−0.0082 (7)
C5	0.0463 (9)	0.0375 (9)	0.0418 (10)	−0.0014 (7)	−0.0062 (8)	−0.0101 (8)
N2	0.0591 (9)	0.0351 (8)	0.0488 (9)	−0.0029 (6)	−0.0070 (8)	0.0017 (7)
C9	0.0379 (8)	0.0318 (8)	0.0336 (9)	−0.0030 (6)	−0.0001 (7)	−0.0033 (7)
C8	0.0397 (9)	0.0324 (9)	0.0376 (9)	−0.0030 (7)	0.0033 (7)	−0.0053 (7)
C13	0.0527 (10)	0.0538 (12)	0.0391 (10)	−0.0031 (8)	−0.0053 (8)	−0.0128 (10)
C12	0.0563 (11)	0.0610 (12)	0.0445 (11)	0.0052 (9)	−0.0124 (10)	0.0009 (11)
C11	0.0582 (11)	0.0405 (11)	0.0574 (12)	0.0063 (8)	−0.0091 (10)	0.0023 (10)
C10	0.0524 (10)	0.0326 (9)	0.0528 (12)	−0.0006 (7)	−0.0056 (10)	−0.0074 (9)
N3	0.0519 (8)	0.0330 (8)	0.0406 (8)	−0.0022 (6)	−0.0058 (7)	−0.0004 (7)
N1	0.0587 (9)	0.0295 (7)	0.0486 (9)	−0.0043 (6)	−0.0009 (8)	−0.0086 (7)
O1	0.0533 (7)	0.0323 (7)	0.0560 (8)	0.0004 (5)	−0.0065 (6)	−0.0008 (7)
O2	0.0630 (7)	0.0339 (7)	0.0542 (8)	0.0015 (5)	−0.0221 (7)	−0.0036 (6)
O3	0.0654 (8)	0.0509 (8)	0.0430 (7)	0.0145 (6)	−0.0147 (6)	−0.0089 (6)

### Geometric parameters (Å, º)

**Table:**

C7—O1	1.2265 (18)	C9—N3	1.371 (2)
C7—O2	1.3102 (19)	C9—C8	1.390 (2)
C7—C4	1.468 (2)	C9—C10	1.404 (2)
C4—C3	1.388 (2)	C8—N1	1.348 (2)
C4—C5	1.396 (2)	C8—C13	1.395 (3)
C3—C2	1.369 (2)	C13—C12	1.364 (3)
C3—H3	0.9300	C13—H13	0.9300
C2—C1	1.381 (2)	C12—C11	1.403 (3)
C2—H2	0.9300	C12—H12	0.9300
C1—O3	1.3545 (19)	C11—C10	1.355 (3)
C1—C6	1.381 (2)	C11—H11	0.9300
C6—C5	1.380 (2)	C10—H10	0.9300
C6—H6	0.9300	N1—H1	0.8600
C5—H5	0.9300	O2—H2A	0.8200
N2—N3	1.3008 (19)	O3—H3A	0.8200
N2—N1	1.339 (2)		
			
O1—C7—O2	121.74 (15)	N3—C9—C10	131.39 (15)
O1—C7—C4	123.97 (15)	C8—C9—C10	120.69 (16)
O2—C7—C4	114.28 (13)	N1—C8—C9	103.95 (15)
C3—C4—C5	118.12 (15)	N1—C8—C13	133.65 (17)
C3—C4—C7	120.59 (15)	C9—C8—C13	122.39 (16)
C5—C4—C7	121.30 (15)	C12—C13—C8	115.53 (17)
C2—C3—C4	121.11 (16)	C12—C13—H13	122.2
C2—C3—H3	119.4	C8—C13—H13	122.2
C4—C3—H3	119.4	C13—C12—C11	122.75 (19)
C3—C2—C1	120.55 (16)	C13—C12—H12	118.6
C3—C2—H2	119.7	C11—C12—H12	118.6
C1—C2—H2	119.7	C10—C11—C12	121.73 (17)
O3—C1—C2	118.07 (15)	C10—C11—H11	119.1
O3—C1—C6	122.67 (15)	C12—C11—H11	119.1
C2—C1—C6	119.26 (16)	C11—C10—C9	116.89 (16)
C5—C6—C1	120.36 (15)	C11—C10—H10	121.6
C5—C6—H6	119.8	C9—C10—H10	121.6
C1—C6—H6	119.8	N2—N3—C9	108.75 (14)
C6—C5—C4	120.56 (15)	N2—N1—C8	111.29 (14)
C6—C5—H5	119.7	N2—N1—H1	124.4
C4—C5—H5	119.7	C8—N1—H1	124.4
N3—N2—N1	108.09 (14)	C7—O2—H2A	109.5
N3—C9—C8	107.91 (14)	C1—O3—H3A	109.5
			
O1—C7—C4—C3	−18.0 (2)	N3—C9—C8—C13	178.84 (16)
O2—C7—C4—C3	161.82 (15)	C10—C9—C8—C13	−1.3 (3)
O1—C7—C4—C5	161.86 (16)	N1—C8—C13—C12	179.32 (19)
O2—C7—C4—C5	−18.3 (2)	C9—C8—C13—C12	0.6 (3)
C5—C4—C3—C2	1.3 (3)	C8—C13—C12—C11	0.6 (3)
C7—C4—C3—C2	−178.86 (17)	C13—C12—C11—C10	−1.2 (3)
C4—C3—C2—C1	−1.3 (3)	C12—C11—C10—C9	0.5 (3)
C3—C2—C1—O3	−179.75 (16)	N3—C9—C10—C11	−179.47 (17)
C3—C2—C1—C6	−0.1 (3)	C8—C9—C10—C11	0.6 (2)
O3—C1—C6—C5	−178.87 (16)	N1—N2—N3—C9	−0.20 (18)
C2—C1—C6—C5	1.5 (3)	C8—C9—N3—N2	0.25 (18)
C1—C6—C5—C4	−1.5 (3)	C10—C9—N3—N2	−179.65 (18)
C3—C4—C5—C6	0.1 (2)	N3—N2—N1—C8	0.09 (19)
C7—C4—C5—C6	−179.74 (16)	C9—C8—N1—N2	0.06 (18)
N3—C9—C8—N1	−0.18 (17)	C13—C8—N1—N2	−178.79 (18)
C10—C9—C8—N1	179.72 (16)		

### Hydrogen-bond geometry (Å, º)

**Table:**

*D*—H···*A*	*D*—H	H···*A*	*D*···*A*	*D*—H···*A*
N1—H1···N2^i^	0.86	2.20	2.982 (2)	152
O2—H2*A*···N3^ii^	0.82	1.87	2.6817 (19)	169
O3—H3*A*···O1^iii^	0.82	1.88	2.6912 (17)	171
C13—H13···O3^iv^	0.93	2.49	3.373 (2)	159

Symmetry codes: (i) −*x*, −*y*+2, *z*+1/2; (ii) *x*+1/2, −*y*+3/2, *z*+1; (iii) −*x*+1/2, *y*+1/2, *z*−1/2; (iv) *x*, *y*, *z*+1.

## References

[bb1] Bruker (2004). *APEX2* and *SAINT* Bruker AXS Inc., Madison, Wisconsin, USA.

[bb2] Dubey, A., Srivastava, S. K. & Srivastava, S. D. (2011). *Bioorg. Med. Chem. Lett.* **21**, 569–573. 10.1016/j.bmcl.2010.10.057 21130647

[bb3] Gaikwad, N. D., Patil, S. V. & Bodade, V. D. (2012). *Bioorg. Med. Chem. Lett.* **22**, 3449–3454. 10.1016/j.bmcl.2012.03.094 22520260

[bb4] Sheldrick, G. M. (1996). *SADABS* University of Göttingen, Germany.

[bb5] Sheldrick, G. M. (2008). *Acta Cryst.* A**64**, 112–122. 10.1107/S0108767307043930 18156677

[bb6] Sieroń, L. (2007). *Acta Cryst.* E**63**, o2089–o2090.

[bb7] Spek, A. L. (2009). *Acta Cryst.* D**65**, 148–155. 10.1107/S090744490804362X PMC2631630 19171970

[bb8] Sudhahar, S., Krishnakumar, M., Sornamurthy, B. M., Chakkaravarthi, G. & Mohankumar, R. (2013). *Acta Cryst.* E**69**, o279. 10.1107/S1600536813001785 PMC3569806 23424552

[bb9] Yang, Y. X., Li, K., Wang, Y. J. & Li, Q. (2010). *Beijing Shifan Dax. Xue. Zir. Kex. (J. B. Norm. Univ.)*, **46**, 160–165.

